# Distribution, sources and toxicity of heavy metals in surface sediments of north western Karnataka, south India

**DOI:** 10.1038/s41598-022-19672-w

**Published:** 2022-09-22

**Authors:** Ishfaq Ahmad Mir, M. SreePrabash, V. Sridhar, K. V. Maruthi

**Affiliations:** 1 Geological Survey of India, State Unit: Karnataka and Goa, Bengaluru, Karnataka 560111 India; 2 Geological Survey of India, Remote Sensing and Aerial Survey Division, Bengaluru, Karnataka 560111 India

**Keywords:** Biogeochemistry, Environmental sciences, Environmental social sciences

## Abstract

This study presents spatial distribution, sources and toxicological risks of As, Cr, Cu, Hg, Ni, Pb, and Zn in the surface sediments from north-western Karnataka, southern India. Heavy metals (except Hg) are 1–5 times enriched than upper continental crust. High concentration of Cr, Ni, Cu, and Zn is in the central Kudalgaon, Devarayi, and Tavargatti and in the south-western Ganeshgudi area, whereas Arsenic is enriched in the north-eastern Alnavar, Kakkeri,Tavargatti and Pb, and Hg in the north-western Kapoli, Devarayi, Manjarpal villages. The ecological risk index, toxic risk index, and mean probable-effects-levels quotient of heavy metals suggest that ~ 40% of the area is prone to very high risk especially for Cr and As to the hydrological, biological, and ecological systems. Multivariate statistical analysis suggests possible geogenic sources for Ni, Cr, Cu, and Zn and anthropogenic sources such as emissions from vehicles and agricultural sectors for As, Hg, and Pb. This study is the first of its kind in the area, which will help, in better formulation of environmental pollution and risk related remedial measures to conserve the natural ecosystem and the well-being of humans.

## Introduction

Essential micro-nutrients are required for the functioning of living beings; however, excess amounts of these elements produce cellular and tissue damage leading to a variety of adverse health effects in humans, plants, and animals^1^. Heavy metals (HMs) like As, Cd, Cr, Pb and Hg^2^ have a higher degree of toxicity, making these 5 metals a top priority for public health concerns. Since the last decade, there is a great awareness in the scientific community about public health and environmental risks associated with HMʹs pollution form increased industrial processes, agricultural activities, combustion, electronic applications, and municipal waste^3,4^. Even at lower levels of exposure, the toxicity of HMʹs is known to induce multiple organ damage.

Surface sediments on planet Earth receive a huge amount of pollutants every year from various geogenic and anthropogenic sources, making soil components not only as a pollutant sink but also act as a buffer by controlling the transport of pollutants in the environment^5,6^. High concentrations of As, Cr, Cd, Hg, and Pb in soil are toxic to most plants and animals.

In the Karnataka state of south India, ~ 16% share in gross domestic production (GDP) is contributed by the agriculture sector, which is higher than the national average^7^. Pesticide and fertilizer use in agriculture fields is the key strategy for controlling crop diseases and improving crop yields. However, in recent years, concern has been growing about the improper usage of hazardous agrochemicals^8^. Agro-chemical overdoses are creating health issues such as skin problems, eye irritation symptoms, breathing problems, dehydration, vomiting, cramps, and diarrhoea in the farming community^7^.

Geochemical mapping was carried out in toposheet no. 48I/11 to prepare a multi-element geochemical database finding its use in mineral resource development, land use planning, agriculture, forestry, environmental monitoring, human and animal health, and proper waste disposal. The present study based on seven heavy metals (As, Cr, Cu, Hg, Ni, Pd, and Zn) from surface sediments provides a broader view of the concentration, distribution, enrichment, sources, and potential environmental risks of these heavy metals.

## Study area

**Figure 1:**
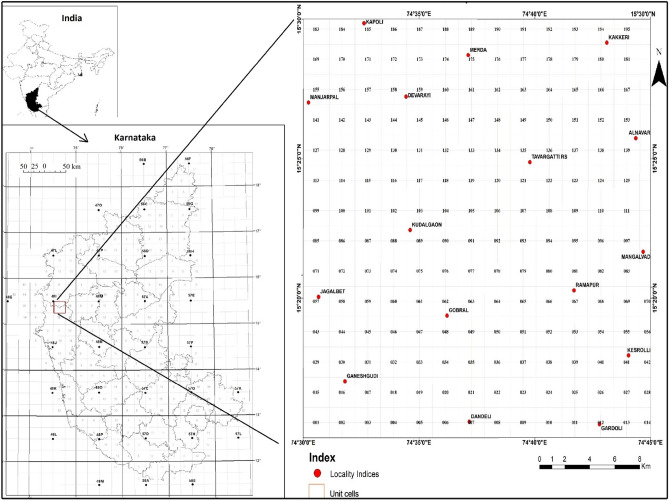
Location map of the study area (Survey of India toposheet no 48I/11) and sampling points. Map prepared using Microsoft Paint and Origin-Pro 2016 software (URL link: http://www.originlab.com).

**Figure 2:**
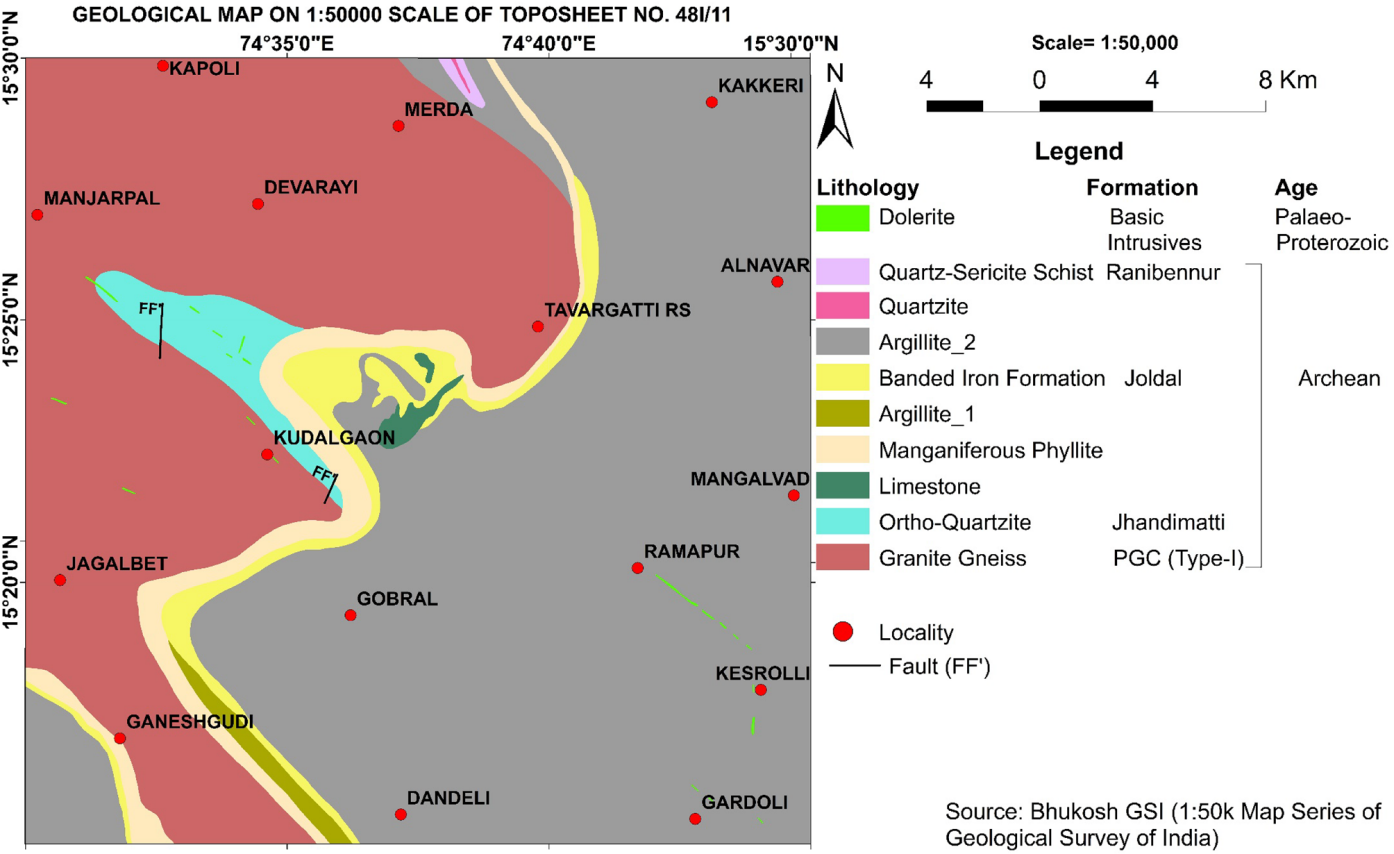
Geological map of the study area, Source: Bhat et al., 2021. Map prepared using Microsoft Paint and Origin-Pro 2016 software (URL link: http://www.originlab.com).

**Figure 3:**
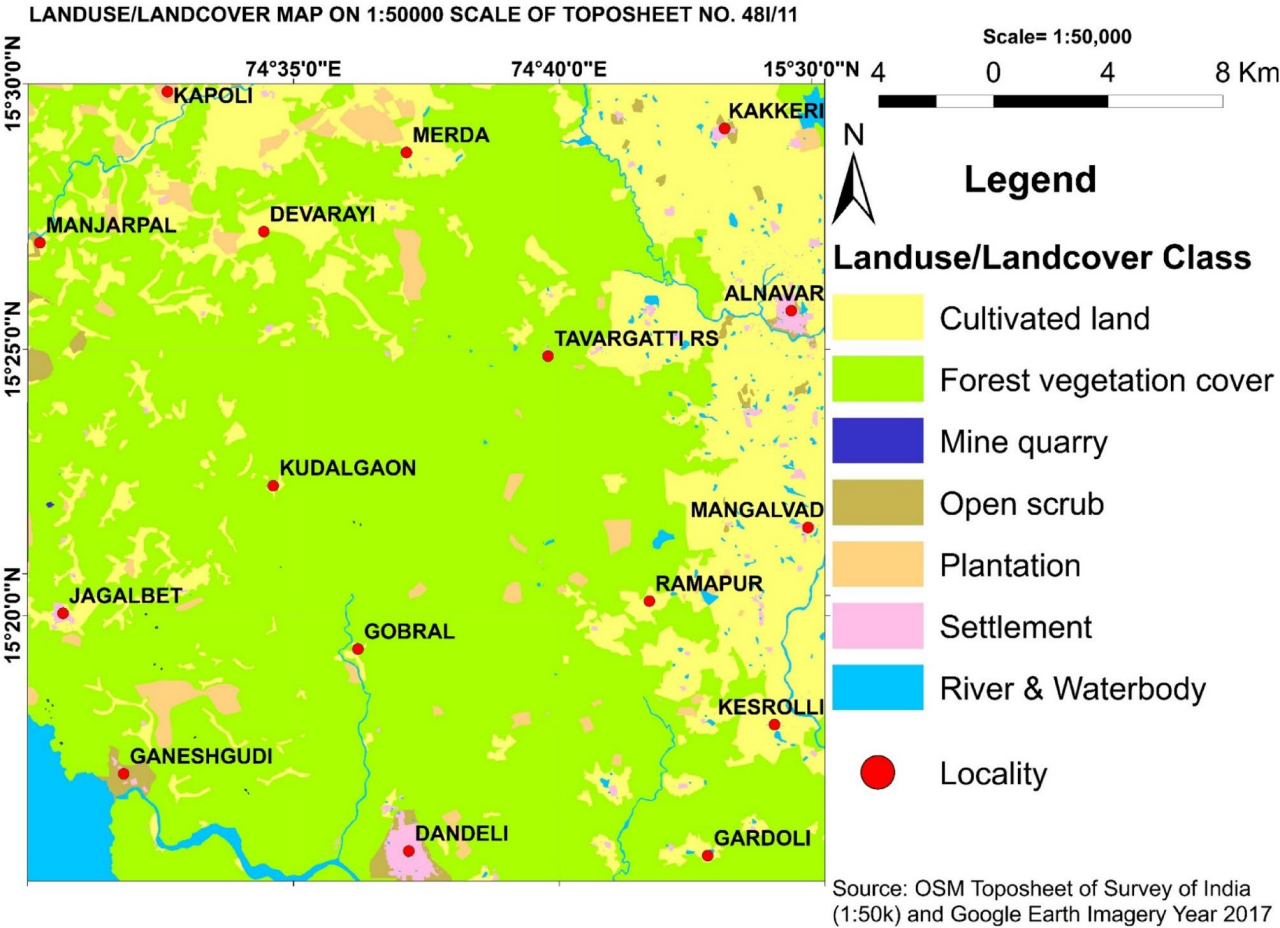
Land-use land-cover map of the study area, Source: Bhat et al., 2021. Map prepared using Microsoft Paint and Origin-Pro 2016 software (URL link: http://www.originlab.com).

The area lies between 15.25° DD to 15.50° DD latitudes and 74.50° DD to 74.75° DD) longitudes (Fig. 1) in the north-western part of Karnataka State, South India, covering parts of Belgaum, Dharwad and Uttar Kannada districts. The western side of the area is hilly terrain with steeply rising hills and narrow valleys. The eastern side is a plain landform, the major drainage pattern of the area is dendritic. The area lies in the Western Dharwar Craton (WDC) of the Indian shield and is occupied by gneissic rocks of the Peninsular Gneissic Complex (PGC); quartzite, limestone, phyllite, argillite, banded iron formation, and schist rocks of Dharwar Supergroup of Shimoga schist belt and dolerite dykes of Younger Intrusives Group. The PGC forms much of the WDC and is made up of tonalitic-trondhjemitic gneiss with many inclusions of older sedimentary and igneous rocks (Fig. 2)^9^. The local climate is mainly warm semi-arid, "BSh class"^10^ and the annual average temperature and rainfall are 24.3 °C and 885 mm respectively. Isolated patches in the entire area and all of the north-eastern part are occupied by human settlements and are under cultivation of sugarcane, rice, cotton, and mango; the remaining part of the area is covered by forests (Fig. 3)^9^.

## Materials and methods

### Sampling no point after “km”

Geochemical mapping on the 1:50,000 scale was carried out by following the standard operating procedure^6,11^ of the National Geochemical Mapping (NGCM) program of the Geological Survey of India (GSI). During field session 2017–2018, in 756 km^2^ areas a total of 752 surface sediments samples (stream sediment and slope wash) on 1 × 1 km^2^ grid pattern and 9 subsoil and duplicate samples on 5′ × 5′ km^2^ grid pattern were collected (Fig. 1). Duplicate samples were collected for cross-check analysis and subsoil samples below 50 cm ground surface were collected for regional background reference. In individual unit cells, fine sediments (silt/clay) were collected within a 100-m distance at a few locations from 1st order streams. Slope wash samples were collected where stream sediments were not available. Samples were sun-dried followed by sieving using −120 size nylon cloth. All samples (< 75 µm grain size) were coned and quartered and 195 composite samples (each representing a 2 km × 2 km grid.) were prepared from the entire toposheet for geochemical analysis.

### Heavy metal analysis

195 stream and slope wash sediment samples were analysed for 10 major oxides (%) and 16 trace elements (ppm) including Cr, Cu, Ni, Pb, and Zn (discussed in this paper) using an X-ray fluorescence (XRF) facility (M/S Pananalytical; MAGIX, 2.4 KW Sequential XRF Spectrometer) at GSI, chemical laboratory, Hyderabad. About 5 g of sample powder (−200 mesh) was spread in the aluminium cup (40 mm diameter in size) over the boric acid powder (AR Grade) and pressed into a pellet under a pressure of 20 tons with the help of a hydraulic press pellet machine to get a uniform pressed pellet. Standard reference material (SRM) (GBW-07312) was analysed after each batch of 20 samples for accuracy and duplicate samples after each batch of 10 samples for repeatability. The accuracy and precision of the measurements were within ± 2%.

Arsenic concentration was measured using a Flow Injection Analysis System-Atomic Absorption Spectrophotometer (FIAS-AAS) instrument at GSI, Chemical laboratory, Hyderabad, (PERKIN-ELMER ANALYST-100). About 0.25 g of sample (−200 mesh) was digested with an acid mixture in the ratio of 2:2:1 ml (HNO_3_ + HClO_4_ + HF) and a few drops of 10% HCl and then evaporated at 180–200 °C to near drying and then again 1:1 HCl was added. SRM (GSD-2a/3a/6) and duplicate samples were also analysed and the accuracy and precision were ± 1%.

Similarly, all samples were analysed for Hg (ppb) concentration using the Direct Mercury Analyser (DMA) instrument at GSI, Chemical laboratory, Hyderabad, (MILESTONE, DMA-80). The samples were ground to −200 mesh particle size and then a 0.2 g sample was weighed into the quartz sample boat and analysed on DMA-80. SRM (GSS-2) and duplicate samples were also analysed and the accuracy was ± 4% and precision was ± 3%.

### Data processing, statistical analysis, and plotting

Mathematical calculations are performed using Microsoft Excel software. Statistical analysis, bivariate plots, location map, geological map, land-use land-cover map and spatial distribution maps are prepared using Microsoft Paint and Origin-Pro 2016 software’s, free download (URL link: http://www.originlab.com).

To evaluate the magnitude of contamination in the surface sediments and their potential bio-environmental risks, four indices were calculated: enrichment factor (EF), ecological risk index (ERI), toxic risk index (∑TRI), and mean probable-effects-levels quotient (M-PEL-Q).

Descriptive statistical parameters (Table 1) such as minimum, maximum, mean, skewness, kurtosis, standard deviation, and variance were computed for stream and slope wash sediments to compare with local sub soil background and the upper continental crust (UCC)^12^ and local background collected from subsoil samples (C) at a depth of 50 cm below ground surface. To determine the relationship between the HMʹs and their probable sources, multivariate statistical analyses were performed. In this study, Pearson’s correlation matrix (PCM, Fig. 7) and hierarchical cluster analysis (HCA, Fig. 7a) were used. The dendrogram is derived from the HCA of heavy metals, the cluster method used is the nearest neighbour, and the distance type is an absolute correlation. PCM and HCA are used to investigate the similarities between element patterns and the provenance of the contaminants.

**Table 1:** Descriptive statistics of the studied heavy metals and their comparisons with upper continental crust (UCC) Taylor and McLennan, (1985) and local background collected from sub soil samples (C) at a depth of 50 cm below ground surface.

Descriptive statistics									
N = 195	UCC	Subsoil (C) Background	Stream and slope wash sediments						
			Minimum	Maximum	Mean	Skewness	Kurtosis	Std. Dev.	Variance
Ni (ppm)	20	79	17	346	50	4.9	32.8	34	1186
Pb (ppm)	20	22	11	56	21	1.6	3.9	7	44
Cr (ppm)	35	201	46	1177	168	5.2	37.6	114	12,936
Cu (ppm)	25	37	10	146	34	3.0	19.2	14	205
Zn (ppm)	71	91	17	179	76	1.5	4.0	24	552
As (ppm)	1.5	4	0.5	36.9	7.6	1.4	2.8	7	47
Hg (ppb)	50	21	9	103	25.6	2.8	12.9	11	125
Fe_2_O_3_ (%)	5	7.2	1.95	23.31	5.8	2.9	12.6	2.7	7.2

## Results

**Figure 4:**
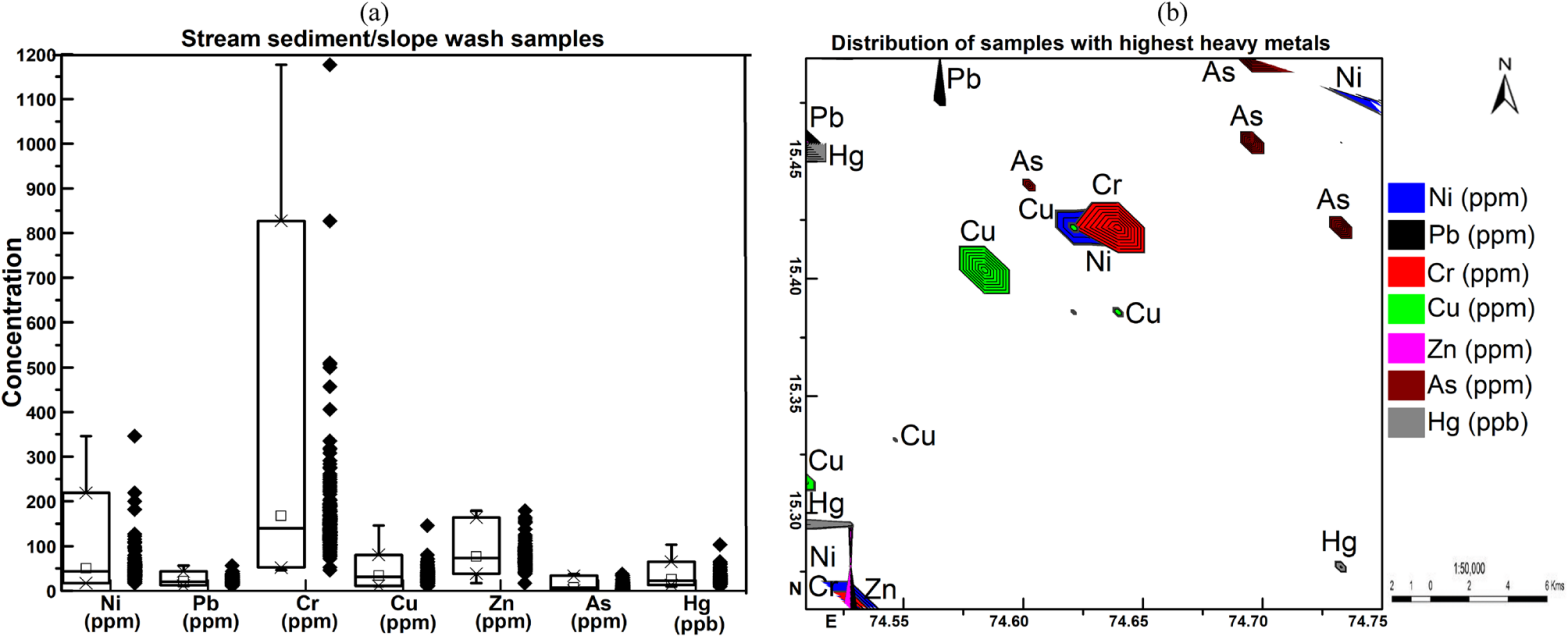
Heavy metal (**a**) concentrations in studied samples, and (**b**) distribution of samples with high metal content in the surface sediment samples of the study area.

The concentration of HMʹs in stream sediments is shown in Fig. 4a and the spatial distribution of samples having the highest content of metals in the entire area is shown in Fig. 4b. Descriptive statistics along with crustal averages and regional background reference are provided in Table 1 (Supplementary Data 1). The absolute concentration of HMs showed 44–12,396 order of variability with an average value of 168, 50, 21, 34, 76, 25.6, and 7.6 ppm for Cr, Ni, Pb, Cu, Zn, Hg, and As respectively. The Cr and As are significantly enriched; Ni is moderately enriched; and Pb, Cu, Zn, and Hg are deficient to minimally enriched in sediments compared to the average upper crust.

Samples from the centre of the study area between Kudalgaon, Devarayi, and Tavargatti villages and in the south-western part near Ganeshgudi village show higher Cr abundance (1177 ppm) (Fig. 2). The distribution of Cr overlaps with Ni and is very similar to the distribution pattern of Cu and Zn (Fig. 4), indicating almost similar origins for these metals. In this area, there is no agricultural or major human activity suggesting the mostly geogenic origin of these metals. The granite gneiss, banded iron formation, and maganiferrous phyllite (Fig. 2) are the major rock types exposed in this area and could be the probable sources of these metals. In contrast, topsoil samples from the north-eastern part of the study area between Devarayi, Merda, Kakkeri, and Alnavar villages (Fig. 4) showed mainly Arsenic enrichment. In this area, there is extensive sugarcane, rice, and mango farming that depends heavily on groundwater for irrigating the farmland. The argillite of Shimoga schist belt and granite gneiss (Fig. 2) are the rock types exposed in this area.

A higher concentration of Pb (56 ppm) and Hg (103 ppb) (Fig. 4) prevailed in the north-western part of the study area between Manjarpal and Kapoli villages (Fig. 2). Granite gneiss (Fig. 2) constitutes the major lithological unit exposed in this area.

## Discussion

### Enrichment factor (EF)

The EF^13^ is calculated by relating the metal (M) concentration in samples with that of Earth’s crust^12^. In this study, Fe is considered as a reference^13^ and EF is calculated by using the following equation:

1 $${\text{EF}} = ({\text{M}}/{\text{Fe}})_{{{\text{sample}}}} /\left( {{\text{M}}/{\text{Fe}}} \right)_{{{\text{crust}}}}$$

**Figure 5:**
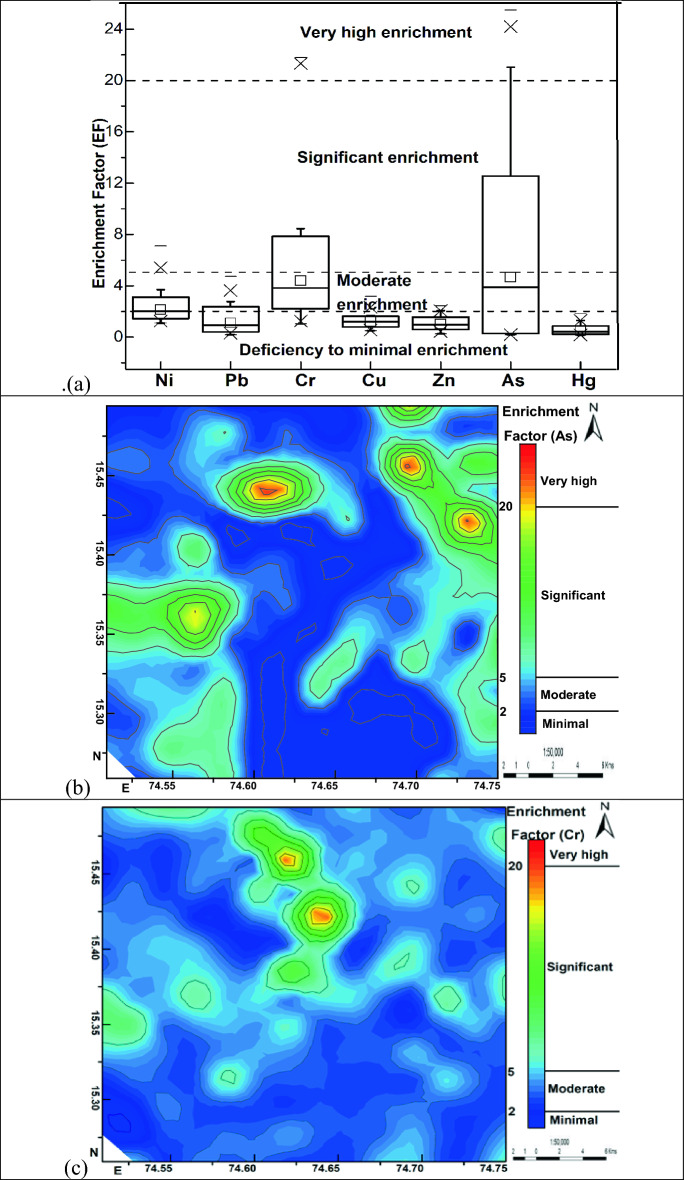
(**a**) Enrichment factor of selected heavy metals, the spatial distribution of enrichment factors for (**b**) As and (**c**) Cr in the surface sediment samples.

The EF is used to discriminate between natural versus anthropogenic sources. The EF value close to 1 generally indicates a geogenic source for HMs and an EF value > 1.5 suggests an anthropogenic contribution from non-crustal materials^14^. EF analysis cannot exactly assess the degree of contamination in the natural environment, it broadly classifies the geogenic or anthropogenic sources of the pollutants and the role of human activities, climate, weathering, erosion, and other sedimentary processes involved in the distribution of the metals^15^. Geochemical surveys indicate that the EF of metals in soils is influenced by various natural and anthropogenic processes and contamination is one of the factors^15^. EF of HMʹs calculated showed low to high enrichment for As (0.18–25.47) and Cr (1.02–21.78), low to moderate enrichment for Ni (1.09–7.09) and Pb (0.18–4.74), and low enrichment similar to crust for Cu (0.49–3.16), Zn (0.24–2.43), Hg (0.15–1.84) suggesting As, Cr and Ni as the major pollutants of concern in the study area. Spatial distribution mapping of EF (Fig. 5) showed significant to very high EF values for As in the north-eastern, northern, and western parts of the study area overlapping with the area under agriculture practices of sugarcane, rice, and mango farming. Excessive application of pesticides and fertilizers for higher crop yield and disease protection and extensive groundwater pumping for irrigation would have resulted in As enrichment. The EF of As shows no overlap with the local geology, indicating the anthropogenic sources of As contamination. On the other hand, significant to very high EF values for Cr in the northern, central, and western parts of the study area overlap with the area covered by granite gneiss rocks. Further, EF of Cr values does not overlap with the agricultural field area, indicating geogenic sources for Cr contamination (granite gneiss). Arsenic and Cr exhibited a weak correlation with each other (0.04, Fig. 7b) confirming different sources of these toxic metals, most probably pesticides, fertilizers, and groundwater irrigation for As and local granite gneissic rocks for Cr.

The distribution of As (ppm) is not matching with lithology (Fig. 4) suggesting a mostly anthropogenic origin. Arsenic-rich pesticides and overexploitation of groundwater are the probable anthropogenic sources of As (ppm) in this area. The distribution of Pb and Hg in the sediments also does not match with lithology (Fig. 4) suggesting a mostly anthropogenic origin for these metals. Ongoing intense illegal mining for construction material in this region suggests a predominant anthropogenic origin for Pb and Hg. Emissions from machines and vehicles used in the extraction of construction material and its transportation could be the probable sources of Pb and Hg.

### Ecological risk index (∑ERI)

The ERI^16^ considers the toxicity of all HMs, ERI of a particular metal (i) is calculated by using the following equation:

2 $${\text{ERI}} = {\text{Tr}}^{{\text{i}}} \times {\text{Cf}}^{{\text{i}}}$$

where Tr^i^ is the biological toxic response factor of metal (i): As = 10, Cr = 2, Cu = 5, Ni = 5, Pb = 5, Zn = 1, Hg = 40)^16^ and Cf^i^ is the ratio between regional geological background and metal measured from surface sediment concentration. The sum of ERI of individual metals is the potential risk (∑ERI) for the water bodies of the area of investigation and is calculated by using the following equation:

3 $$\sum {\text{ERI}} = \sum \left( {{\text{Tr}}^{{\text{i}}} \times {\text{Cf}}^{{\text{i}}} } \right)$$

**Figure 6:**
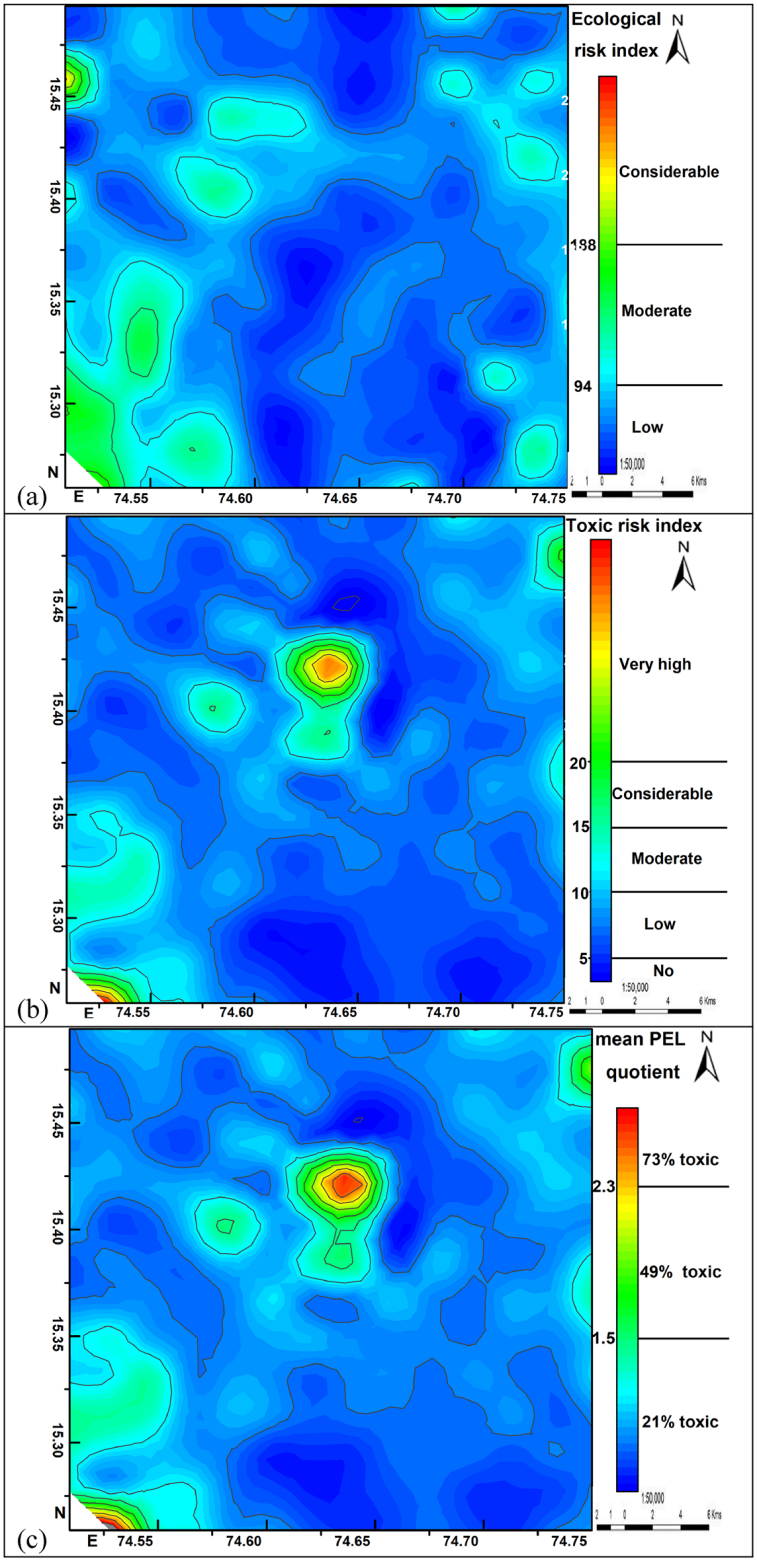
Spatial distribution of potential risks assessed by (**a**) ecological risk index, (**b**) toxic risk index, and (**c**) mean probable-effects-levels quotient methods.

In this study, ∑ERI presented is calculated according to the terminology used by^17^. The ∑ERI is calculated from the individual ERI of 7 metals of the surface sediments. ∑ERI^17^ distribution in the study area is calculated from the summation of individual ecological risks^16^ of Ni, Pb, Cr, Cu, Zn, As, and Hg (Fig. 6a). The individual ERIʹs plots do not give a better toxicity picture of the area. To overcome this problem, a quick and simple method ∑ERI is used for evaluating the level of pollution^18^. This method classifies the study area into different levels of contamination zones and identifies the high toxic area for necessary actions^19^. This method despite being developed for lacustrine systems is still widely used by researchers on land pattern contaminations also^17–19^. The model is formulated based on simple algorithms and has an organized structure including the mathematical relationships between the environmental parameters. The ∑ERI is based on the heavy metal chemistry of surface sediments from the surroundings of aquatic systems. Surface sediment sampling and their chemical analysis are easier than water and are more representative of spatial and temporal distribution. The sediment geochemical data provides a high concentration of contaminants with higher stability than water thereby reducing the possibility of errors due to detection limits of the analytical methods applied.

∑ERI is considerable (~ 200) in about 5% of the area, moderate (~ 94–200) in about 35% of the area and is low (~ 38–94) in about 60% of the area. Water bodies (Supa dam reservoir and Kali river) around Ganeshgudi-Jagalbet villages in the south-western part, around Devarayi-Manjarpal villages in the north-western part, and Kakkeri-Alnavar-Mangalvad villages in the north-eastern part of the study area shows a moderate to considerable level of potential ecological risk for the aquatic system and their biota. The dense forest stretch from north to south between Merda-Dandeli villages (Fig. 2) in the middle part of the study area is almost free from human encroachment and anthropogenic activities. The distribution of low ∑ERI values overlaps with this dense forest area. The highest values of ∑ERI around Ganeshgudi village could be due to the Supa dam reservoir-related anthropogenic activities and around Alnavar village could be due to the settlement-related anthropogenic activities such as vehicle emissions, agriculture, construction, and domestic waste. The low-risk zone in the middle dense forest area is habituated by a few tribal villages that live in close harmony with nature.

### Toxic risk index (∑TRI)

The TRI^20^ considers the threshold-effect-level (TEL) and probable-effects-levels (PEL) of metals to assess the biological risks. The individual toxic risk index (TRIi) is calculated by using the following equation:

4 $${\text{TRIi}} = \surd ({\text{C}}_{{\text{s}}}^{{\text{i}}} /{\text{C}}_{{{\text{TEL}}}}^{{\text{i}}} )^{2} + ({\text{C}}_{{\text{s}}}^{{\text{i}}} /{\text{C}}_{{{\text{PEL}}}}^{{\text{i}}} )^{2} /2$$

where C^i^_s_ is the surface sediment metal concentration, and C^i^_TEL_, and C^i^_PEL_ are the TEL and PEL of the metals respectively. TEL for As = 5.9, Cr = 37.3, Cu = 35.7, Ni = 18, Pb = 35, Zn = 123, Hg = 0.17 and PEL for As = 17, Cr = 90, Cu = 197, Ni = 26, Pb = 91.3, Zn = 315, Hg = 0.486 ^20^. The sum of individual TRIi of metals is the potential toxicity risk index (∑TRI) for the sediments and is calculated by using the following equation:

5 $$\sum {\text{TRI}} = \left( {{\text{TRIi}}1 + {\text{TRIi}}2 + \ldots {\text{TRIi}}7} \right)$$

The toxic units (TUs) of HMs consider only the probable effect level (PEL)^21^ and ignores the threshold effect level (TEL) to calculate the ecological toxicity. Using only PEL values, this equation underestimates the ecological toxic risk, to overcome this problem^20^ developed a new toxic risk index (TRI) integrating both TEL and PEL based toxic levels of HMs in the surface sediments and their potential risks to the aquatic organisms (Eq. 4). The individual toxic risk index (TRIi) of metals in this study are significantly variable for Ni (0.81–16.53), Pb (0.23–1.21), Cr (0.94–24.15), Cu (0.20–2.94), Zn (0.10–1.10), As (0.063–4.69) and Hg (0.039–0.45). TRIi in the sediments is below 5 for Pb, Cu, Zn, As and Hg suggesting the absence of any toxic risk, and above 5 for Ni and Cr indicating the presence of toxic risk of these metals to the aquatic life. The ecological toxicity classification based on integrated toxic risk (∑TRI, Eq. 5) values is shown in Fig. 6b, which summarized the toxic risk index to provide a better picture of toxicity risks. The ∑TRI in the study area varies from 3.45-to 38.44, and ~ 60% of the area has ∑TRI < 5 indicating no toxicity, and the remaining ~ 40% of the study area has a low to very high risk of toxicity. Spatially the ∑TRI of the HMʹs is highest in the central (Kudalgaon-Tavargatti-Devarayi region), south-western (Ganeshgudi region), and north-eastern (Kakkeri area) (Fig. 6b). Chromium and Ni are the highest contributors, whereas As, Cu, Pb, Zn, and Hg have the lowest contribution to ∑TRI. Contaminated sediments in high toxic risk areas need to be given more attention due to their high contributing ratios to ∑TRI.

### Mean probable-effects-levels quotient (M-PEL-Q)

The M-PEL-Q^22^ considers the mean probable-effects levels (PEL) to determine the biological effects and potential ecological risk of the HMs. The M-PEL-Q is calculated by using the following equation:

6 $${\text{M - PEL - Q}} = ({\text{C}}^{{\text{i}}} /{\text{PEL}}^{{\text{i}}}_{1} + {\text{C}}^{{\text{i}}} /{\text{PEL}}^{{\text{i}}}_{2} + \ldots {\text{C}}^{{\text{i}}} /{\text{PEL}}^{{\text{i}}}_{7} )$$

where C^i^ is the surface sediment metal concentration, and PEL^i^_1…0.7_ are the PEL of the metals discussed respectively, PEL is same as mentioned in Eq. (4).

The quality of topsoil accessed by the mean probable-effects-levels quotient method (M-PEL-Q)^22^ is a useful tool to decipher the impact of heavy metal pollution on aquatic life^23^. The M-PEL-Q uses the combined effects of heavy metals (Eq. 6) considering PEL values to calculate the potential biological and ecological risks. The M-PEL-Q values in the study area vary from 0.26–3.5 (Fig. 6c), indicating HMʹs are posing 21–73% toxic effect on the hydrobiota. Spatially ~ 50% of the area is having moderate (21%), ~ 30% of the area is having significant (49%), and ~ 20% of the area is having high (73%) chances of toxicity risk (Fig. 6c). The M-PEL-Q value of < 1.0^24^ is usually considered to be safe and does not require any action to be taken. In this study, M-PEL-Q values for the entire area vary from 0.26 to 3.5, with ~ 60% of the area having an M-PEL-Q value < 1.0 which is under the safe limit. The remaining ~ 40% of the area has an M-PEL-Q value between 1.0–3.5 which is above the safe limit, and can pose moderate to high (21–73%) biological and ecological risks which need urgent action to prevent further deterioration. The M-PEL-Q and ∑TRI^20^ have similar distribution patterns suggesting that both methods are acceptable tools for the assessment of biological and ecological toxicity risks for sediments, vegetation, aquatic and terrestrial organisms, and human beings.

Based on the results of these four assessment methods, ~ 40% of the surface sediments, water bodies, and the ecology in the Belgaum, Dharwad, and Uttar Kannada districts of Karnataka state India can be described as having moderate to high pollution risk posed by HMs and requires urgent attention of policymakers and the general public from further environmental and human health deterioration. Anthropogenic sources of contamination such as excessive use of pesticides, fertilizers, automobile emissions, and over-exploitation of groundwater need to be reduced. The area with the high potential for biological, hydrological, and environmental risks caused by anthropogenic contamination is mainly concentrated in the north-eastern agriculture belt surrounding the Alnavar village and in the south-western area surrounding Ganeshgudi village near Supa dam reservoir. The rest of the study area is having low to moderate toxic risks and is in the safe zone. Arsenic and Cr are the high-risk toxic elements, Ni is the considerable risk toxic element, and Pb, Cu, Zn, and Hg are the low-risk toxic elements in the study area. Chromium and Ni contamination is broadly contributed by natural processes and Arsenic contamination is mainly contributed by human activities. The ∑TRI and mean probable-effects-levels quotient^22^ showed a good correlation with each other suggesting that the TRI is an acceptable accurate method for the assessment of ecological toxicity.

### Identifying the sources of metals

**Figure 7:**
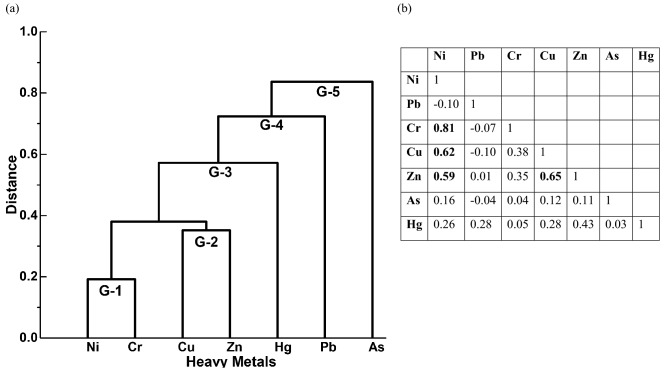
(**a**) Hierarchical cluster analysis and (**b**) Pearson’s correlation matrix of selected heavy metals in the surface sediment samples used in this study, bold values indicate strong correlation of metals with each other.

Multivariate statistical analysis methods of hierarchical cluster analysis (HCA, Fig. 7a) and Pearson’s correlation matrix (PCM, Fig. 7b) are performed to identify the origin, similar behavior, and distribution of HMʹs in the study area. HCA identified five groups (G1-5) of association between seven HMʹs (Fig. 7a) used in this study. G-1 consisting of Ni and Cr, and G-2 consisting of Cu and Zn indicate that these metal groups are derived from similar sources. G-3 consists of Hg, G-4 consists of Pb and G-5 consists of Arsenic suggests that these metal groups are derived from different sources. Probable sources of groups G-1 (Ni = 17 to 346 ppm and Cr = 46 to 1177 ppm) seems to be derived from gneissic rocks and for G-2 (Cu = 10 to 146 ppm and Zn = 17 to 179 ppm) metals from Joldal and PGC Formations due to the similarity in distribution pattern with local geology. G-3 (Hg = 9 to 103 ppb), G-4 (Pb = 11 to 56 ppm), and G-5 (As = 0.5 to 36.9 ppm) are possibly originating from anthropogenic sources as their higher concentrations overlap well with the land-use distribution but do not overlap with the geology of the area. Hg and Pb seem to be originating from atmospheric emissions of automobiles, and agricultural machines. High Arsenic content in sediments of the north-eastern side is consistent with large-scale sugarcane plantation and excessive groundwater pumping for better crop yield. Arsenic-rich pesticides and overexploitation of groundwater are therefore possible sources of Arsenic contamination in the surface sediments. The use of pesticides, fertilizers, and other anthropogenic activities will contribute to the heavy metal pollution in the Belgaum, Dharwad, and Uttar Kannada districts of Karnataka state, but are beyond the scope of the present study. Correlation analysis (PCM) also indicates a similar observation. Ni, Cr, Cu, and Zn showing a strong positive correlation (0.59–0.81) with each other suggests similar geological sources, and a weak correlation with Hg, Pb, As (−0.04–0.26) suggests different sources. Hg, Pb, and As further show a weak correlation (−0.04–0.28) with each other indicating different anthropogenic sources.

## Conclusions

Heavy metal study in the surface sediments was conducted in the Belgaum, Dharwad, and Uttar Kannada districts of Karnataka, India to decipher the level of contamination and potential biological and ecological risks. The average concentration of studied metals is higher than crustal averages except for Hg. It is evident that the topsoil samples in the studied region exhibit significant to very high enrichment for As and Cr and minimum to moderate enrichment for Ni, Pb, Cu, Zn, and Hg. Higher concentrations of Cr, Ni, Cu, and Zn were observed in and around the central and south-western part, whereas Arsenic in the north-eastern part and Pb, and Hg in the north-western part of the study area. The application of the ecological risk index, toxic risk index, and mean probable-effects-levels quotient suggest elevated contents of toxic metals (Ni, Pb, Cr, Cu, Zn, As, Hg) which are posing moderate to high hydrological, biological, and ecological risks in ~ 40% of the area and low to moderate risks in rest of the area. Hierarchical cluster analysis deployed for source identification revealed possible geogenic sources for Ni, Cr, Cu, and Zn and anthropogenic sources for As, Hg, and Pb. Arsenic is possibly contributed by As rich pesticides and overexploitation of groundwater for irrigation purposes. Mercury and Pb possibly are contributed by atmospheric emissions coming from automobiles and agriculture machines. The correlation matrix of heavy metals also provides a similar picture to that of cluster analysis. In summary, it can be concluded from observed results that surface sediments in the studied region are highly contaminated by Cr and As and moderately contaminated by Ni, Pb, Cu, Zn, and Hg. Since this is the first heavy metal pollution study on surface sediments in the Belgaum, Dharwad, and Uttar Kannada districts of south India, more detailed studies are required to obtain adequate knowledge of the contamination levels and their potential risks for better land and water management and sustainable policies formulation.

## Supplementary Information

Supplementary Information.

## Data Availability

All data generated or analysed during this study are included in this published article (and its Supplementary Information files).
